# Relative importance of AMH and androgens changes with aging among non-obese women with polycystic ovary syndrome

**DOI:** 10.1186/s13048-015-0175-x

**Published:** 2015-07-09

**Authors:** Vitaly A. Kushnir, Noy Halevy, David H. Barad, David F. Albertini, Norbert Gleicher

**Affiliations:** Center for Human Reproduction, 21 East 69th Street, New York, NY 10021 USA; Wake Forest School of Medicine, Winston-Salem, NC USA; Sackler School of Medicine, Tel Aviv University, Tel Aviv, Israel; Foundation for Reproductive Medicine, New York, NY USA; University of Kansas, Lawrence, KS USA; Rockefeller University, New York, NY USA

**Keywords:** Polycystic ovarian syndrome (PCOS), Ovarian reserve, Androgens, Testosterone, Anti-Müllerian hormone (AMH)

## Abstract

**Background:**

To assess the changes in phenotypes and endocrine profiles of women with polycystic ovary syndrome (PCOS) with advancing age.

**Methods:**

In a cross-sectional study conducted at a private tertiary fertility clinical and research center we identified anonymized electronic records of 37 women who had presented with a prior diagnosis of PCOS. They were stratified as younger (<35 years) and older (≥40 years). As controls, we identified 43 women with age-specific low functional ovarian reserve and 14 young women with normal functional ovarian reserve. Endocrine profiles for each group were evaluated based on total (TT) and free testosterone (FT), anti-Müllerian hormone (AMH) and sex hormone binding globulin (SHBG).

**Results:**

Patients including those with PCOS were mostly non-obese, evidenced by normal BMIs (21.6 ± 6.0) with no differences between study groups. Young PCOS patients presented with a typical pattern of significant hyperandrogenemia and elevated AMH in comparison to young women with normal functional ovarian reserve [TT 44.0 (32.9–58.7) vs. 23.9 (20.3–28.1) ng/dL, (*P*<0.05); and AMH 7.7 (6.2–9.1) vs. 2.5 (2.0–3.0) ng/mL, (*P*<0.05)]. With advancing age, hyperandrogenemia in PCOS diminished in comparison to young women with normal functional ovarian reserve, resulting in similar TT levels [28.6 (19.7–37.5) vs. 23.9 (20.3–28.1) ng/dL]. Though also declining, AMH remained significantly elevated in older PCOS women in comparison to young women with normal functional ovarian reserve [4.0 (2.7–5.2) vs. 2.5 (2.0–3.0) ng/mL, (*P*<0.05)]. Patients with low functional ovarian reserve demonstrated significantly lower AMH at both young and older ages compared to women with normal functional ovarian reserve (*P*<0.05 for both). However, among patients with low functional ovarian reserve no differences were observed at young compared to older ages in TT [17.6 (12.9–24.1) vs. 18.1 (13.6–24.1) ng/dL)] and AMH [0.4 (0.3–0.6) vs. 0.3 (0.2–0.5) ng/mL]. SHBG did not differ significantly between groups but trended opposite to testosterone.

**Conclusions:**

The PCOS population predominantly consisted of non-obese phenotype at both young and advanced ages. This suggests that patients with “classical” obese PCOS phenotype rarely reach tertiary infertility care, while non-obese PCOS patients may be more resistant to lower levels of infertility treatments. PCOS patients also demonstrate more precipitous declines in testosterone then AMH with advancing age. These data support incorporation of AMH as diagnostic criterion for PCOS regardless of age, and imply that testosterone should not be relied upon in the diagnosis of PCOS in older women.

## Background

The polycystic ovary syndrome (PCOS) is an amalgam of clinical conditions, to a large degree characterized by a polycystic ovary phenotype (POP) [1]. Various classifications have been proposed to define the syndrome, consensus opinions have been issued [2–7], though none have found universal acceptance. The 2003 Rotterdam criteria for diagnosis of PCOS are, likely, the currently most widely accepted definition of PCOS, including irregular ovulatory function (oligomenorrhea or amenorrhea), evidence of hyperandrogenism (chemical or clinical) and presence of an POP on sonography [2].

Principal reasons for lack of consensus are likely differing etiologies and pathophysiologies leading to PCOS [8]. In addition, increasing evidence suggests that PCOS is not stable and/or static as women advance in age [9].

Many of the typical phenotypic features, especially hyperandrogenism and anovulation, normalize with advancing age [10]. Consequently, women, who may present with fairly typical PCOS at young ages by older ages, when they reach fertility treatments, may no longer exhibit those typical findings. Though their history of PCOS may still have clinical relevance, their PCOS diagnosis may no longer be obvious to treating physicians.

Importance of normal androgen levels for normal follicle growth and maturation and, therefore, for female fertility has been increasingly recognized over the last decade [11]. Declines in androgen levels with advancing age [10, 12], therefore, have the potential of affecting ovarian functions and female fertility. Consequently, androgen supplementation has been utilized in hypoandrogenic infertile women with low functional ovarian reserve (LFOR) [13].

Because PCOS ovaries (and their androgen receptors) may from younger years be used to higher androgen levels, functional hypoandrogenism may be comparatively more pronounced in older PCOS patients than normal older women.

Further complicating diagnosis and classifications of PCOS is that only a fraction of patients exhibit the “classic” PCOS phenotype, characterized by trunkal obesity (high BMI) [4]. A similar percentage of women with PCOS presents without obesity and may also lack other phenotypical characteristics of “classic” PCOS [14, 15].

Whether high BMI and non-obese low BMI PCOS patients differ in their respective ovarian aging patterns is still unknown. Because both start from different endocrine and metabolic baselines, it appears possible that they differ in how they evolve with advancing age.

Androgens characteristically decline with age [12]. In patients with LFOR they are, however, comparatively low at all ages [16]. PCOS patients, in contrast, at young ages are almost uniformly hyperandrogenic. Hyperandrogenemia, however, usually resolves with advancing age, at times allowing for spontaneous resumption of regular menstruation at relatively advanced reproductive ages [4]. Interestingly, despite similar androgen levels, young non-obese PCOS patients demonstrate fewer signs and symptoms of hyperandrogenism than “classical” PCOS patients. This is likely the consequence of increased bio-availability of androgens in peripheral tissues and enhanced 5α-reductase activity in obese patients [17].

Whether different PCOS phenotypes “age” differently has so far not been studied. Should there be differences, they might have significant clinical consequences. One very obvious one would be the clinical accuracy of diagnosis of PCOS at older ages, based on Rotterdam criteria.

We recently proposed that, functionally, and in hormonal parameters, PCOS and LFOR represent opposing extremes of ovarian function, characterized by hyper- and hypo-androgenemia and hyper- and hypoactive follicle recruitment, respectively [18]. Like in PCOS, the natural history of LFOR over time is, however, largely unknown.

This study, therefore, aimed to improve the understanding of androgen dynamics in women with PCOS and LFOR over advancing age. In completing this study, we gained interesting and, at times surprising, new insights into the non-obese PCOS phenotype. They point toward potential new treatment options, and also allow for the development of new hypotheses about the pathophysiology of PCOS.

## Methods

### IRB and informed consents

All patients at our center sign at initial consultation an informed consent, which allows use of their medical record data for clinical research as long as their identity remains protected and the medical record remains confidential. Since both of these conditions were met, this study qualified for expedited review and approval by the center’s IRB.

### Patient populations

All 94 patients investigated in this cross sectional study presented to our fertility center between 2009 and 2014, and were part of an anonymized electronic research database our center has been maintaining. Among those, 37 were study subjects, prior to presentation to our center diagnosed elsewhere with PCOS. To qualify for this study group, they in addition to on medical records review having been formally diagnosed with PCOS, had to have verified histories of oligo/amenorrhea consistent with intermittent anovulation and female infertility.

Two distinct patient groups, during the same time period treated at our center, served as controls. The first control group included 43 women with LFOR, with the diagnosis defined by anti-Müllerian hormone (AMH) below age-specific 95 % CI, as previously reported for our center’s patient population [19, 20]. Under age 35 years such patients were classified as premature ovarian aging (POA), by some also given the acronym occult primary ovarian insufficiency (oPOI). Above age 40 years, we consider all patients with LFOR to suffer from physiologic ovarian aging. A second control group involved 14 young women with normal functional ovarian reserve (NFOR), who during the study period had entered fertility treatment for either male factor infertility or tubal disease. These patients were used to establish normal reference values, reflecting young, normal ovaries.

PCOS and LFOR groups were further stratified by age into younger (age <35 years) and older (age ≥40 years) groups, thus yielding a total of five distinct study groups (Table 1).

**Table 1:** Patient characteristics and baseline hormone measurements

	Young	Older	Young	Older	Young
	PCOS	PCOS	LFOR	LFOR	NFOR
N	21	16	24	19	14
Age (years)	30.7 (29.3–32.1)	41.6 (40.9–42.4)*	32.9 (31.8–34.1)	41.7 (41.0–42.4)*	31.4 (29.6–33.1)
BMI (kg/m^2^)	21.6^a^ (19.1–24.6)	23.5 (20.4–26.5)	20.8 (18.8–22.7)	24.0^a^ (21.9–26.3)	21.1 (17.7–24.4)
AMH (ng/mL)	7.7 (6.2–9.1)*	4.0 (3.3–4.8)*	0.4 (0.3–0.6)*	0.3^a^ (0.2–0.5)*	2.5 (2.0–3.0)
Total Testosterone (ng/dL)	44.0^a^ (32.9–58.7)*	27.8^a^ (21.8–35.5)	17.6^a^ (12.9–24.1)	18.1^a^ (13.6–24.1)	23.9^a^ (20.3–28.1)
Free Testosterone (pg/mL)	4.1 (2.2–5.9)*	1.5^a^ (1.0–2.2)	0.7^a^ (0.4–1.1)	1.4 (0.9–1.9)	1.6 (1.2–2.0)
DHEA (ng/dL)	398.8 (223.3–574.3)	269.6^a^ (205.1–354.4)	315.3 (223.3–407.3)	274.6^a^ (189.2–398.5)	302.8 (211.6–394.1)
DHEA-S (ug/dL)	236.6 (170.6–302.5)	183.9 (124.4–243.5)	130.6 (99.3–171.8)	158.5^a^ (117.1–214.5)	231.9 (157.3–306.4)
SHBG (nmol/L)	71.0 (26.7–115.2)	86.5 (56.9–116.1)	76.0^a^ (56.6–102.0)	65.6^a^ (50.4–85.4)	75.4^a^ (52.2–109.0)

Data in this table are reported as Mean (95 % Confidence Interval)

^a^indicates data back-transformed after initial logarithmic transformation

*indicates statistically significant difference (*P* < 0.05) in comparison to Young NFOR group

### Laboratory testing

All laboratory tests were performed at time of initial presentation to our center, when patients undergo a first diagnostic evaluation. AMH and serum androgens were performed at random, unrelated to day of menstrual cycle. Laboratory tests were performed via commercial testing (Laboratory Corporation of America). AMH was measured by enzyme-linked immunosorbent, Gen II assay (Beckman Coulter, Inc., Webster, Texas). All androgen levels were measured by liquid chromatography/tandem mass spectrometry.

### Statistical analyses

Statistical analyses were performed in MedCalc, Version 14.8.1. (Ostend, Belgium). Normality of data was tested for all continuous variables. Those not normally distributed were log transformed. Values are, where appropriate, shown as means and 95 % confidence intervals (CI). One-way ANOVA was used to evaluate differences between study groups. Post-hoc analysis was performed with Student-Newman-Keuls (SMK) test for pair wise comparisons. A *P*-value <0.05 was considered statistically significant.

## Results

Table 1 summarizes patient characteristics, including baseline hormone measurements for all study groups. Significant age differences between older and younger patient groups were a feature of study design. However, distribution of age was similar within respective young and older subgroups. Surprisingly, BMI values were practically identical between all five patient groups.

**Fig. 1:**
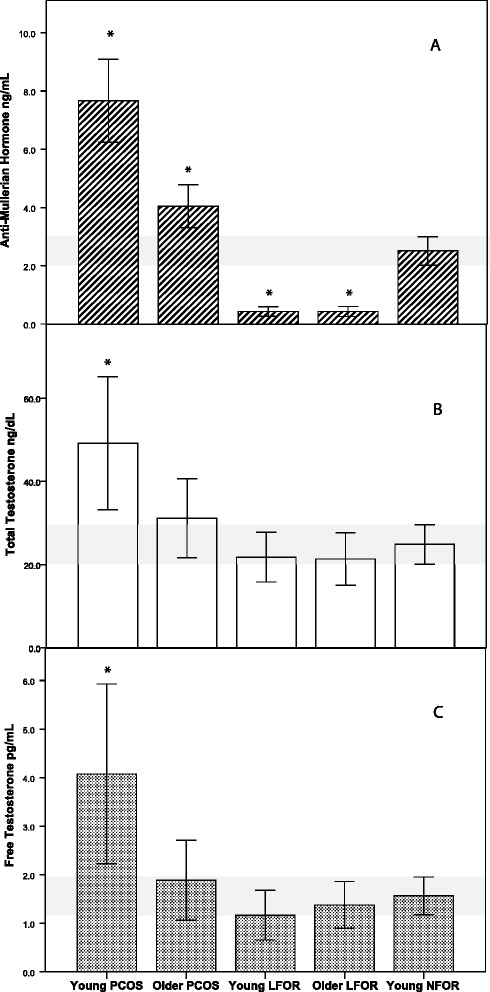
AMH and testosterone levels in women with PCOS and LFOR stratified by age. **a** AMH levels (ng/mL). **b** Total Testosterone (ng/dL). **c** Free Testosterone (pg/mL); Error bars represent 95 % Confidence Intervals. Shaded area corresponds to the 95 % Confidence Interval of the Young NFOR comparison group. *indicates statistically significant difference (*P* < 0.05) in comparison to Young NFOR group

As demonstrated in Fig. 1, young PCOS women presented with a typical pattern of significantly elevated AMH and hyperandrogenism in comparison to young women with NFOR [TT 44.0 (95 % CI, 32.9 to 58.7) vs. 23.9 (95 % CI 20.3 to 28.1) ng/dL, (*P* < 0.05); AMH 7.7 (95 % CI 6.2 to 9.1) vs. 2.5 (95 % CI 2.0 to 3.0) ng/mL, (*P* < 0.05)]. With advancing age, hyperandrogenemia in association with PCOS, however, diminished significantly (*P* < 0.05), resulting in similar TT and FT levels to young women with NFOR [TT 27.8 (95 % CI 21.8 to 35.5) vs. 23.9 (95 % CI 20.3 to 28.1) ng/dL].

AMH, however in older PCOS women remained significantly elevated in comparison to young women with NFOR [4.0 (95 % CI 3.3 to 4.8) vs. 2.5 (95 % CI 2.0 to 3.0) ng/mL, (*P* < 0.05)]. As expected, AMH was significantly lower in older than younger PCOS women (*P* < 0.05; Fig. 1).

Younger and older LFOR patients demonstrated significantly lower AMH levels in comparison to young women with NFOR [0.4 (95 % CI 0.3 to 0.6) and 0.3 (95 % CI 0.2 to 0.5) respectively vs. 2.5 (95 % CI 2.0 to 3.0) ng/mL] (both *P* < 0.05). Interestingly, AMH levels did not decrease in older LFOR in comparison to young LFOR patients, possibly because even young LFOR patients already approached lower levels of AMH assay sensitivity [21].

This explanation, however, does not hold up for androgens: as previously reported [16], TT and FT levels even in young LFOR patients trended lower (though, likely due to relatively small sample size, did not reach significance) in comparison to young controls with NFOR. Yet, TT and FT in older women with LFOR also did not further decrease in comparison to young LFOR women [18.1 (95 % CI 13.6 to 24.1) vs. TT 17.6 (95 % CI 12.9 to 24.1) ng/dL], suggesting that whatever causes relative hypoandrogenemia in these patients at already young ages appears to mimic the physiologic hypoandrogenemia of older age. In other words, LFOR at young ages truly appears to reflect “premature ovarian aging”.

Since androgen effects on follicle development are primarily mediated by T (via androgen receptor) [11], it is not surprising that DHEA and DHEAS levels were similar among all the groups. Sex hormone binding globulin (SHBG) trended into the opposite direction to T but did not reach statistical significance (Table 1).

## Discussion

Despite relatively small patient numbers in all five patient groups, this study produced surprisingly robust statistical data. Likely, the most remarkable finding of the study was the recognition that in identifying established PCOS patients who had sought out fertility treatment at our center, practically all were of the “non-classical,” non-obese phenotype.

The study allows for this conclusion for a number of reasons: Based on very high AMH levels and hyperandrogenemia at young ages, here investigated young infertility patients, unquestionably, had been correctly assigned a diagnosis of PCOS. That their BMI was practically identical to the BMI of young controls with NFOR precludes the possibility that this group of PCOS patients to a significant degree represented the “classical” PCOS phenotype.

Infertile women who reach tertiary fertility centers like ours have not only failed to spontaneously conceive but, in most cases, also failed to conceive with first line ovulation inducing medications, like clomiphene citrate and aromatase inhibitors, mostly administered by general gynecologists at earlier infertility treatment stages. The finding that PCOS patients referred to our tertiary fertility center were predominantly non-obese, therefore, defines here investigated PCOS patients as different from average PCOS populations, generally described in the literature [22].

That PCOS patients reaching tertiary infertility care at our center practically exclusively turned out to be non-obese PCOS patients, therefore, suggests that this PCOS phenotype already at young ages may be more resistant to fertility treatments than “classical” PCOS, who likely conceive with lower levels of care.

If confirmed by other studies, this here for the first time reported observation would suggest distinctly different underlying pathophysiology for these two distinct PCOS phenotypes. It would also confirm a recent report from Azziz’s group, which suggested that referral biases affect the prevalence of obesity in PCOS patient populations [22].

As Fig. 1 demonstrates, the non-obese PCOS phenotype loses between young and older age, based on AMH levels, approximately 50 % of functional ovarian reserve (FOR). Yet, FOR still remains above what even at young ages is considered a NFOR. Androgens, however, follow a different trajectory: as Fig. 1 demonstrates, TT and FT also decline and, indeed, proportionally decline less than AMH. TT and FT levels, yet, approximated the normal range of young women with NFOR and, indeed, no longer differed statistically.

As AMH is produced in granulosa cells of growing follicles [23], these findings suggest that, considering still ongoing excessive follicle recruitment (i.e., still high AMH levels), non-obese PCOS patients at older ages produce relatively deficient amounts of T in ovarian theca cells and/or adrenals, even though in young controls with NFOR, these T levels would be considered in entirely normal range.

Women with non-obese PCOS, therefore, comparatively suffer from relative hypoandrogenemia because of asynchrony between growing follicle volume and androgen levels. Based on this observation, it is tempting to hypothesize that the reason why women with “classical” PCOS may conceive easier (and earlier) may be that their more pronounced hyperandrogenism may prevent such asynchrony, and the resulting relative hypoandrogenemia with advancing ages, from occurring.

Here observed rather remarkable differences between PCOS and LFOR patients offer further insights into the interplay between growing follicular cell mass and androgen levels. In contrast to significant declines in FOR (i.e., AMH) and androgens (TT and FT) in non-obese PCOS patients, women with LFOR demonstrated practically no detectable changes in both parameters between younger and older ages. They, thus, at both age extremes exhibit a hormonal profile of cellular exhaustion.

This observation strongly supports the contention that, hormonally, POA at young ages presents with a very similar profile to physiological ovarian aging at older age, and explains why androgen supplementation in women with LFOR appears similarly effective in younger and older women [13].

Androgens induce PCOS-like POPs in various animal models [24]. They work synergistically with FSH during small growing follicle stages by enhancing the sensitivity of granulosa cells to follicle stimulating hormone (FSH) [25]. A detailed review on the subject was recently published [11]. We demonstrated that combining androgen supplementation with consecutive FSH exposure in sequential in vitro fertilization cycles improves FOR and improves oocyte yields at advanced ages even in women with severe LFOR [26].

What initiates hyperandrogenism in association with PCOS and relative hypoandrogenism in association with LFOR has so far remained unresolved. We have hypothesized about the existence of a yet undiscovered androgen production factor (APF) of, possibly, immune system origin, which is hyperactive in association with PCOS and hypoactive in LFOR [18].

Here presented data also suggest potential for new clinical therapies. For example, if non-obese PCOS patients despite seemingly normal androgen levels, indeed, suffer from relative hypoandrogenism, as has been reported in LFOR patients [13, 27], androgen supplementation may help in improving oocyte/embryo quality and fertility treatment outcomes.

Here reported data should also lead to better diagnosis of non-obese PCOS in older infertile patients, a diagnosis currently widely overlooked. At young ages, AMH and androgens easily differentiate women with PCOS and LFOR from women with NFOR. At older ages, the differential diagnosis, as Table 1 and Fig. 1 demonstrate becomes significantly more complex.

While TT and FT levels statistically differentiated young PCOS patients from women with LFOR and NFOR, these markers, as here demonstrated, become inadequate at older ages. In contrast, AMH levels appear to maintain their differentiating power even into older age, therefore allowing for the identification of PCOS regardless of age.

AMH, therefore, in PCOS women of advanced reproductive age should be considered a better disease marker than T, supporting the incorporation of AMH levels into updated criteria for the diagnosis of PCOS at all ages [28]. Conversely, T should not be relied upon in the diagnosis of PCOS in older women.

## Conclusions

These data suggest that the “classical” PCOS phenotype with elevated BMI only rarely reaches tertiary infertility care. If confirmed, non-obese PCOS patients may be more resistant to lower levels of infertility treatments. They with advancing age also demonstrate more precipitous T than AMH declines. Low and high BMI PCOS patients may, therefore, reflect distinctively different pathophysiologies. These data also suggest that AMH at all ages is a better diagnostic criterion for PCOS than T.

## Abbreviations

PCOS: Polycystic ovary syndrome
LFOR: Low functional ovarian reserve
NFOR: Normal functional ovarian reserve
TT: Total Testosterone
FT: Free testosterone
AMH: Anti-Müllerian hormone
SHBG: Sex hormone binding globulin
POP: Polycystic ovary phenotype
BMI: Body mass index
oPOI: Occult primary ovarian insufficiency
CI: Confidence intervals
SMK: Student-Newman-Keuls
APF: Androgen production factor
FSH: Follicle stimulating hormone
T: Testosterone
